# Migrant families’ experiences of participating in the Family Talk Intervention when affected by childhood cancer

**DOI:** 10.2340/1651-226X.2025.44811

**Published:** 2025-12-10

**Authors:** Maria Ayoub, Malin Lövgren, Maja Holm, Camilla Udo

**Affiliations:** a School of Health and Welfare, Dalarna University, Falun, Sweden; b Department of Health Care Sciences, Palliative Research Centre, Marie Cederschiöld University, Stockholm, Sweden; c Advanced Pediatric Home Care, Astrid Lindgren Children’s Hospital, Karolinska University Hospital, Stockholm, Sweden; d Department of Nursing Sciences, Sophiahemmet University, Stockholm, Sweden

**Keywords:** The Family Talk Intervention, migrant families, paediatric care, psychosocial support

## Abstract

**Background and purpose:**

The psychosocial needs of migrant families affected by a child’s severe illness are extensive. However, few family-centred interventions have been evaluated and even fewer have included families with migrant backgrounds. The aim of this study was, therefore, to explore migrant families’ experiences of participating in a family-centred psychosocial intervention, the Family Talk Intervention (FTI), in a paediatric care setting.

**Material and methods:**

In this study, semi-structured interviews were performed with 14 family members (six parents, one ill child, and seven siblings) after participating in FTI. The interviews were transcribed and analysed using thematic network analysis.

**Results:**

After participating in FTI, the families experienced that, in their already exposed situation, their family stability had increased as they were supported in dealing with social and financial issues, encouraged to talk openly about difficulties, and thus became closer as a family. Both children and parents described the value of having someone professional, continuously available, to turn to for guidance and information.

**Interpretation:**

Migrant families dealing with a child’s severe illness live in an exposed situation, with a double burden of distress related to the child’s illness and socioeconomic factors. By acknowledging the importance of these families’ psychosocial needs, it could be recognised that psychosocial support, such as FTI, not only aids family adjustment but also contributes to reducing this double burden, increasing family stability.

## Introduction

Coping with a child’s severe illness is one of the most stressful life-changing events for both the ill child and the whole family. In this study, severe illness refers to conditions that can be classified as life-threatening or life-limiting, such as cancer [1]. The psychosocial sequelae of the child’s illness, and its impact on family life, are explained by altered family dynamics and relationships due to the stressful situation, financial difficulties, uncertainty about the future, and the emotional burden on all family members [2–5]. When considering these illness-related stressors, it is important to acknowledge that certain factors, such as migrant backgrounds, cultural differences, and lower socioeconomic status, might represent a double burden for the family and their psychosocial adjustment [5, 6].

Increased global migration has led to many families either moving with their children or becoming parents in a new country [7]. For instance, in Sweden approximately every third resident has at least one parent with a non-European background, with Arabic-speaking individuals representing the largest group [8]. Many migrant families in Sweden also navigate a prolonged and uncertain residence process, often involving temporary permits and repeated renewals [9]. Considering this worldwide migration, new challenges have arisen in paediatric care, such as managing language barriers and different cultural backgrounds [10, 11]. Migrant families in paediatric care have described unfamiliarity with the healthcare systems in the new country and unmet needs for support [12, 13]. To overcome such barriers, and to meet the families’ needs, studies have stressed the importance of access to professional interpreters [14, 15]. However, despite using interpreters in paediatric care, cultural and language barriers still exist, contributing to disparities in access to social and emotional support [14, 16, 17]. Addressing these inequities is crucial since strong evidence suggests that psychosocial support plays a significant role in treatment success and family adjustment when a child is impacted by a severe illness [18–20]. Psychosocial support alongside medical treatment is needed to successfully help them deal with the difficult family situation [21, 22].

One family-centred psychosocial intervention is the Family Talk Intervention (FTI), originally developed for use in psychiatric care [23]. FTI includes the whole family and aims to facilitate family communication about illness-related subjects, support parenting, and help families in identifying their strengths and how to use them [24]. FTI has been pilot-tested in a paediatric oncology context in Sweden [25] showing improved family communication, increased illness-related knowledge, and strengthened family togetherness [26, 27]. However, until now, studies on FTI have been conducted in a homogenous sample in paediatric oncology among Swedish-speaking families [26, 28]. Migrant families with severely ill children may face unique psychosocial burdens. As worldwide migration increases diversity, also in paediatric care, it is important to include these families when evaluating support. The aim of this study is, therefore, to explore migrant families’ experiences of participating in FTI in a paediatric care setting.

## Material and methods

### Design

This qualitative study was conducted in paediatric care with migrant families having a child who had a severe illness, an understudied population. Interview data from parents, ill children, and siblings were used to explore these families’ experiences of participating in FTI. The study is reported according to the criteria recommended by the Standards for Reporting Qualitative Research (SRQR) Checklist.

### The FTI

**Figure 1:**
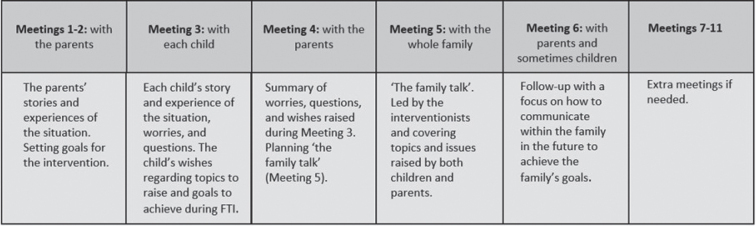
Family members involved and the contents of each meting in the Family Talk Intervention (FTI).

FTI is a manual-based intervention consisting of six 1-hour meetings with family members in various constellations (Figure 1). These meetings are led by an FTI-educated healthcare counsellor, often called hospital social workers (HSW). Although the length of the intervals between meetings is flexible and adapted to each family, they are often held at 1–2-week intervals.

### Study setting and participants

Eligible families were those with a migrant background having a child who had a severe illness [1] being cared for at a university hospital in Sweden. Parents in the participating families had to be foreign-born, with no requirement to speak or understand Swedish. The whole family or part the family could participate in FTI, but at least one parent and one child/youth (ill or healthy) aged 0–19 years had to be included. HSWs conducting the intervention identified families that met the inclusion criteria, provided them with study information and asked them about participation. The recruitment process was pragmatic to facilitate easier participation in these families which otherwise are a hard-to-reach population.

In total, 19 family members from three nuclear families (6 parents, 3 ill children, and 10 siblings) completed the intervention, using a telephone interpreter. The families had two to six children between 3 and 20 years. Two of the ill children were diagnosed with cancer, and one with another severe illness. The families were at different stages of the residence process in Sweden, ranging from recent arrivals to longer-settled households. Of the 19 participating family members, 14 were interviewed (6 parents, one ill child, and 7 siblings) after the intervention completion Those not interviewed were children under the age of 6 years or those who were too ill.

### Data collection

Semi-structured interviews were performed with all family members in the families’ homes according to their preferences. The interviews were conducted by researchers who had no experience working clinically with FTI. Family members were interviewed at the same time but in different rooms. All participants were given the opportunity to use an interpreter in their native tongue, but the majority (12/14) preferred to speak Swedish.

An interview guide was used with separate versions for parents and children. The interview guide initially covered how the family dealt with difficult life events and their experiences of family management and communication. It then focused on their experiences of FTI, including its structure and content, the use of an interpreter, and positive or negative aspects of participation. The same themes were explored with both parents and the children, but the questions were simplified and adapted to the child’s age. Child interviews were conducted with sensitivity and flexibility by researchers experienced in interviewing children. The interviews lasted between 20 and 46 minutes and were audio-recorded.

### Data analysis

The interviews were transcribed verbatim and analysed with thematic network analysis [29]. Thematic network analysis is suitable for analysing textual data into thematic networks that illustrate and summarise the text’s main themes. It allows a rich exploration of the text’s underlying patterns and emerging themes [29]. In the first step of the analysis, each interview transcript was read several times to facilitate data familiarisation. Meaningful text segments were highlighted and extracted into a document. The text segments were then coded by the first author. In the next step, the codes with similar meanings were grouped into basic themes and then clustered into organising themes. Finally, one overarching global theme, representing the core of the analysis, was identified. To enhance credibility and dependability, the analysis was conducted transparently through frequent discussions among all authors. Credibility was maintained through the stepwise process, going back and forward between the analytical stages. To further strengthen credibility, every step in the analysis has been described in detail. Interpretations of data were discussed until consensus was reached, with the authors reflecting on their own pre-understanding to maintain reflexivity. To facilitate transferability, detailed descriptions of the study context, participants, and data collection were presented. Interview data were securely managed and stored according to university research data management routines.

### Ethical considerations

The study was approved by the Regional Ethical Review Board in Stockholm (No. 2020-06341 and 2022-01949-02). Written informed consent, translated into Arabic, were collected from all family members. In accordance with Swedish law, written consent was obtained from parents of children under 15 years, while those aged 15 years and older gave their own written consent. The families were written and verbally informed about the aim of the study and confidentiality. Furthermore, due to the small sample size and the risk of being identified, no detailed information regarding the participants and the child’s illness is described.

## Results

**Figure 2:**
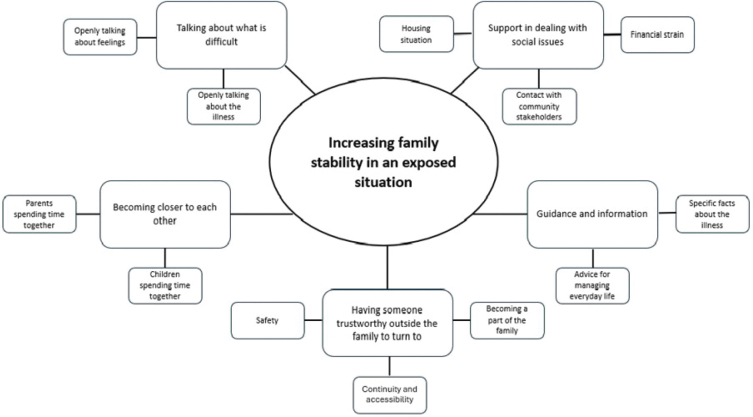
Network of global, organising, and basic themes describing migrant families’ experiences of participating in the Family Talk Intervention (FTI).

From the analysis, a thematic network was developed. Five organising themes, created from the basic themes, constituted one global theme which captures the families’ experiences of participating in FTI (Figure 2). The term ´children´ is used collectively, referring to both ill children and siblings, as this reflects how the families described their experiences.

### Increasing family stability in an exposed situation

Overall, the parents and children in the families experienced that the stability in their family had increased after participation in FTI. Family stability involved descriptions of how FTI supported the parents and children in dealing with social issues related to their housing situation, financial strain, and in contact with various community stakeholders, such as healthcare and welfare services. Participation in FTI also contributed to family members becoming closer because they were encouraged to talk about what was difficult**,** e.g. illness-related issues or how they were feeling. Having someone trustworthy outside the family to turn to for guidance and information also contributed to family stability.

### Support in dealing with social issues

Parents described an exposed situation, not only because of the child’s diagnosis, but also because of social and economic challenges that impacted the whole family. FTI was described as supportive in addressing concerns about temporary residence permits, with parents expressing constant stress over deportation and their children’s future: ‘*this uncertainty, this worry about the temporary residence permit and about [the ill child’s] illness. […] I don’t know what is going to happen*’ (Father). The housing situation, related to overcrowding and a lack of private space, was also addressed during FTI, which was experienced as very helpful by both parents and children.

According to parents and children, participation in FTI helped the families with financial difficulties. The parents described how the interventionist assisted them with an application for financial aid and transportation assistance. One parent stated that such support contributed to them being able to pay the rent, the electricity bill and to buy food: ‘*She [the interventionist] has fought hard. In two weeks, she has sorted everything. We were able to pay the rent*’ (Mother).

Parents and children reported that the interventionist assisted them in their contact with healthcare and social services and the school. This was particularly valuable in supporting siblings’ needs related to schooling and their psychological wellbeing. The siblings described that the interventionist was fighting for them to receive extra social support at school, which had resulted in better school performance and fewer attention problems.

### Having someone trustworthy outside the family to turn to

During FTI, both parents and children said that the interventionist became part of the family, described as a friend, cousin, or even an extra parent, as one child explained: ‘*She [the interventionist] feels like my second mother*’ (Ill child). The children further stated that the interventionist’s continuous home visits became a natural part of the family’s everyday life. As one child illustrated, ‘*Now we are used to her [the interventionist] coming. It’s not strange when she is here. It’s like when a cousin comes to visit us*’ (Sibling). Parents and children repeatedly mentioned feeling safe from knowing that there was someone outside the family who would always help, support, and listen to them.

According to the children, letting an unfamiliar person into the family was not always a matter of course for their parents. However, all parents stated that they completely trusted the interventionist to meet the children alone: ‘*I don’t trust anyone, and if it had been anyone other than [the interventionist] then I wouldn’t have let them [the children] meet her*’ (Mother).

Meeting the interventionist, and having continuous contact, created over time a bond of trust and familiarity. Both children and parents shared that they appreciated the accessibility of the interventionist during FTI, and they did not hesitate to make contact for advice and support if needed. However, a main concern expressed was the uncertainty they felt after FTI had ended and the loss of support and security from the interventionist.

### Talking about what is difficult

Parents shared that they usually had not talked about the diagnosis and prognosis with their children before taking part in FTI to protect them from being worried or afraid: ‘*We don’t talk about certain problems with or in front of the children. These are problems that belong to adults, not minors*’ (Mother). This was also evident in the children’s descriptions: ‘*She [mother] doesn’t usually talk about it [the illness], because she doesn’t want us to be scared*’ (Sibling). At the same time, the children said they often hid their true feelings from parents and siblings for the same reason. However, both children and parents described that FTI created a space in which all family members could gradually reveal their concerns. Using an interpreter during FTI was not perceived as a concern or an issue.

The parents described that FTI made them aware of the importance of talking about difficult feelings, both within the family but also with the interventionist, generating a sense of relief: ‘*We opened up to each other and I realized what it really felt like, thanks to [the interventionist]. Sometimes it feels better and lighter in your heart when you open up and tell someone else*’ (Father).

Although parents rarely talked about the illness with their children, it became clear during FTI that the children understood more than expected, as one child said: ‘*They [the younger siblings] actually understand very well. They may not get angry, but they know a lot more than anyone thinks. So, they know what it is, cancer*’ (Sibling). The children shared that talking openly about the diagnosis with their parents and the interventionist calmed their worries.

### Becoming closer to each other

Parents described having to prioritise caring for the ill child, which left less time for the healthy siblings, also mentioned by some of the siblings. Sometimes it could be hard to do activities together as a family because of the illness. However, according to both parents and children, participation in FTI had brought the family members closer to each other.

At the beginning of FTI, parents and children wanted to improve the children’s relationships with each other. One child shared that he had a close relationship with his sibling when they were younger but, following the sibling’s diagnosis, their relationship had changed. However, participation in FTI supported them in becoming closer as a family: ‘*These past months I’ve become a bit closer to him [brother]*’ (Ill child). This view was also shared by their parents.

Parents described how the child’s diagnosis affected their relationship negatively, especially during intensive treatment periods when they were separated between the hospital and home. However, participation in FTI was reported to have encouraged the parents to find time for each other: ‘*We go shopping together, something we haven’t done before. And we talk a lot. Or drink coffee on the balcony. We do things. Before [FTI] we didn’t do anything together*’ (Mother).

### Guidance and information

The children shared that receiving information and specific facts about their sibling’s diagnosis was valuable for them as this contributed to a sense of control. However, too much information could sometimes be overwhelming: ‘*They explain so much and then you don’t understand. I want them to show things like this with pictures and stuff*’ (Sibling). Parents perceived that the siblings’ increased awareness about the diagnosis fostered understanding and acceptance of the parents’ need to focus on the ill child and sometimes treat the siblings differently.

Parents further mentioned how they sometimes had to confront difficulties while caring for the ill child, who they described could be irritable and moody. The interventionist gave the family advice on how to act. One child described that the interventionist told them that ‘*the best thing we can do for [the ill child] is not be harsh*’ (Sibling), which felt good to know for everyone concerned.

Guidance could also relate to parents needing advice about how to help the siblings open up about jealousy. During FTI, the parents described receiving information and advice from the interventionist about how to deal with such situations, which provided them with ‘moral support’.

## Discussion

This study provides important new knowledge about migrant families’ experiences of participating in a psychosocial support intervention, FTI, in paediatric care settings. In the context of FTI, the families highlighted an exposed life situation, with the emotional burden of chronic stress related to the child’s illness, as well as socioeconomic factors and simultaneously navigating an uncertain future. It has been shown that a family’s migration status can delay seeking care and support and cause emotional distress such as fear of deportation and of family separation [30]. This demonstrates an urgent need to address the psychosocial burden faced by families with a migrant background [13, 21]. Importantly, the results indicate that FTI contributed to meeting these families’ social, financial, informational, and relational needs, so that they are better equipped to deal with the child’s severe illness.

Many of the psychosocial challenges that migrant families in this study experienced are well-documented among all families dealing with a child’s severe illness, regardless of ethnicity [4, 31, 32]. However, the results show that migrant families face challenges not only due to the child’s illness, but also from living conditions, such as limited social networks and economic resources, which impose further distress. When basic needs go unmet, this could potentially distract from achieving some of the more advanced family needs, such as strengthening family relationships. In the present study, it seems that the initial focus in FTI was on meeting the families’ basic needs, such as receiving financial and social support. These findings differ from previous studies of FTI involving their Western counterparts, which were more centred around supporting family functioning, including family communication and relations [26, 27, 33].

The isolation of migrant families dealing with a child’s severe illness, far away from their wider social support network, has been shown to be an important source of distress [34]. Additionally, unfamiliarity with the healthcare and welfare systems in the host country has been identified as a common barrier to utilisation of social support [35], leaving these families more vulnerable. In this study, families valued having a professional who could inform and guide them through various support systems. Furthermore, both children and parents perceived that the continuous and easy access to the interventionist during FTI contributed to a sense of stability in their challenging life-situation. This is consistent with previous research, showing that a trustful relationship with healthcare professionals can decrease language barriers and improve communication to better support migrant families [36], promoting equity and minimising disparities in paediatric care [16, 37].

Hiding information and communication avoidance in families dealing with a child’s severe illness may seem more natural in some cultures, which can limit families’ ability to interact effectively with healthcare professionals and utilise support [22, 38, 39]. In this study, both parents and children stated that support through FTI helped them talk more openly about sensitive topics related to the child’s illness. Even though there was initially some hesitancy among the parents to receive outside support, they appreciated opening up about their worries to someone professional outside the family. The migrant families reported similar results in relation to their experiences of FTI, such as improvements in family communication and relations, as Swedish-speaking families [26, 27, 33]. This indicates that FTI can provide a structured way to address migrant families’ often extensive psychosocial needs in paediatric care, offering both practical and family communication support. In future studies of FTI and other psychosocial support interventions, it is crucial to enable participation of families of diverse backgrounds to learn about their needs so appropriate support can be offered.

### Strengths and limitations

Families with migrant backgrounds are significantly underrepresented in paediatric research and several barriers to recruiting this population into studies have been reported, including those focused on psychosocial interventions [40]. This study, therefore, contributes much needed knowledge. However, the predominantly positive experiences reported by the families must be considered, and may reflect that FTI was the only family-centred support offered, or that families may have been reluctant to report negative aspects due to fear of consequences. Using an interpreter during the interviews allowed participants to respond in their native language and express their experiences of participating in FTI. On the other hand, using an interpreter could have influenced the findings since the language spoken was not that of the researchers. To address this, a native Arabic-speaker connected to the research team has validated the accuracy of the interpreter. Another limitation is the small sample size and the inclusion of only Arabic-speaking families, which limits transferability to other migrant populations. However, it should be kept in mind that this study is a first attempt to broaden FTI to other groups than only Swedish-speaking families.

## Conclusion

Providing family-based psychosocial support to migrant families facing a child’s severe illness can play a crucial role in addressing their often urgent needs. By alleviating these burdens, families are better equipped to manage the child’s illness and cope with its impact despite dealing with e.g. uncertainty of whether to receive permanent residence permit. This study suggests that FTI contribute not only to improved family communication but also to increased stability supporting families exposed to multiple psychosocial stressors. This study highlights the need to consider families within their broader social context, recognising that structural factors may impact on how challenges of a child’s illness are coped with.

## Supplementary Material



## Data Availability

The data are not publicly available due to confidentiality restrictions.
